# Of Headlamps and Marbles: A Motivated Perceptual Approach to the Dynamic and Dialectic Nature of Fairness

**DOI:** 10.1177/20413866231199068

**Published:** 2023-09-06

**Authors:** Michael R. Bashshur, Laurie J. Barclay, Marion Fortin

**Affiliations:** Singapore Management University, Singapore; 3653 University of Guelph, Canada; TSM-Research, CNRS, University Toulouse Capitole, France

**Keywords:** fairness, justice, motivated cognition, Brunswikian lens approach, subjectivity

## Abstract

How do people perceive fairness? Recently, fairness scholars have raised important theoretical questions related to *what* information is used in fairness perceptions, *why* this information is emphasized, and *how* fairness perceptions can change over time. Integrating the Brunswikian lens approach with a motivated cognition perspective, we develop the Motivated Perceptual Approach (MPA) to highlight how people can be motivated to selectively perceive and weight cues to form fairness perceptions that align with their motives. However, these motives can change over time and through interaction with motivated others. By illuminating the *dynamic* and *dialectic* processes underlying fairness perceptions, the MPA sheds light on how people's fairness perceptions can be influenced by their own motives as well as socially constructed and negotiated through interactions with motivated others. Practical insights include how to effectively manage fairness perceptions over time and across perspectives. We conclude with a research agenda for advancing the fairness literature.

**Plain Summary:**

Whether or not people perceive they (or others) have been treated fairly or are treating others fairly at work, has implications for a variety of important outcomes ranging from helping others (when people perceive fairness) to undermining supervisors, making plans to quit or punishing bad actors (when people perceive unfairness). Important questions remain, however, around how people come to these perceptions in the first place. How do they decide what is fair? A long time assumption has been that these perceptions are subjective and motivated; that “fairness is in the eye of the beholder.” Based on this assumption, two people who experience the same event may come away with very different fairness perceptions. This is a crucial insight that helps explain the significant disparities in perceptions of fairness between people. However, as a field, we seem to have strayed from that foundational assumption. In this paper, we revisit this premise to develop an approach describing how people collect and integrate information to inform their fairness perceptions, highlighting the particular role that their motives (what they want to perceive, e.g., that they are fair actors, that they are treated well by important others) shape what information they attend to and use in arriving at their perceptions of fairness. From this perspective we explain how fairness perceptions can change over time, explain and predict differences between perspectives (e.g., managers and employees), and provide guidance for developing practical interventions that can reduce these differences before they become intractable.

“*There is a sense in which all cognition can be said to be motivated. One is motivated to understand the world, to be in touch with reality, to remove doubt, etc…motives like wanting to find the truth, not wanting to be mistaken, etc., tend to align with epistemic goals in a way that many other commitments do not*.”—Harris (2010)

Over the past 50 years, an impressive volume of studies has highlighted the profound and pervasive importance of fairness perceptions for employees and organizations (for meta-analytic reviews, see Colquitt et al., 2013; Rupp et al., 2014).^1^ Importantly, a key assumption within the fairness literature has been the notion that “fairness is in the eye of the beholder” (see Greenberg et al., 1991). That is, people may have vastly different perceptions of fairness, even when they are participating in the same situation. However, the contemporary fairness literature has been heavily criticized for diverting focus away from this key assumption. For instance, Rupp et al. (2017) compellingly argued that the fairness literature is too narrowly focused on a subset of objective justice criteria (“justice rules”) and suggested that “scholars may have collectively succumbed to reification by treating our evolved operationalization of organizational justice as though it represents the actual phenomenon of experiencing justice” (p. 940). Similarly, Barclay et al. (2017) argued that the fairness literature should be regrounded on the theoretical premises that fairness is a subjective and motivated phenomenon. This suggests that implicit assumptions in the contemporary literature may need to be challenged, including notions that everyone has access to the same information or that people perceive/interpret fairness information in the same way. Similarly, considering the broader social context and dynamic interplay between parties may provide deeper insight into how fairness perceptions are socially constructed and negotiated.

Grounding on the premise that fairness perceptions are subjective and motivated, we develop the Motivated Perceptual Approach (MPA) to highlight the temporal and social nature of fairness perceptions. We draw on insights from the Brunswikian lens approach (Brunswik, 1952), which outlines how people collect and use information (i.e., cues) to inform their perceptions. Moreover, we integrate these insights with a motivated cognition perspective (e.g., Kunda, 1990), which highlights how people's motives can influence how they select, evaluate, and weigh the information that ultimately shapes their perceptions. In doing so, the MPA sheds light on how fairness perceptions are a *dynamic* (i.e., fairness perceptions can change as motives change) as well as a *dialectic* phenomenon (i.e., fairness perceptions can be socially constructed and negotiated through interactions with motivated others).

We make three primary theoretical contributions. First, we identify and outline the subjective and motivated perceptual processes underlying fairness perceptions. When people are viewed as subjective and motivated processors of information, their perceptions can be influenced “not solely by the information available in [the] environment but rather by how it relates to whatever goal [is] currently [being pursued]” (Bargh & Chartrand, 1999, p. 446). That is, people are motivated to select and weigh information in a way that suits their motivations, which can change over time. By identifying the motivated perceptual processes underlying fairness, the MPA provides a roadmap for understanding how fairness perceptions develop and change.

Second, the MPA provides a theoretical foundation for understanding the influence of perspective (e.g., actor, recipient, and observer) on fairness perceptions. By highlighting the role of motives and how these may differ depending on one's perspective, it becomes clear *how and why* parties to the same fairness interaction can hold dissimilar fairness perceptions despite having the same underlying perceptual process. The MPA also outlines how each party's perspective can impact the motives that are activated as well as the information (i.e., cues) that is available and how this information is used. In doing so, the MPA provides novel insights for predicaments of unfairness (i.e., when managers, employees, and/or third parties disagree about what is fair; Bies, 1987), including how these predicaments can emerge, when and why some predicaments become intractable, and how predicaments can be prevented or effectively managed when they do occur.

Third, the MPA highlights the importance of considering how fairness perceptions can be socially constructed and negotiated between motivated parties. This includes how the temporal and social nature of interactions can influence fairness perceptions by activating dialectic processes in which parties can actively influence each other's perceptions through implicit or explicit discourses. We propose that fairness perceptions can be dynamically influenced by interplay between the parties, including attempts by one party to persuade the other of the validity of their fairness perceptions (e.g., by expanding cue sets and activating specific motives).

Overall, our objective with the MPA is to highlight the profound insights related to the dynamic and dialectic processes that can emerge when theorizing is grounded on the assumptions that fairness is a subjective and motivated phenomenon. In doing so, the MPA advances a temporal theory of fairness (i.e., how fairness perceptions can unfold and change over time), integrates the literature to account for differences between perspectives (e.g., actors, recipients, and observers), and provides guidance for developing practical interventions that can reduce these differences before they devolve into predicaments of unfairness (i.e., disagreements about what is fair; see Bies, 1987). Finally, given the renewed appreciation for the subjective and dynamic nature of fairness perceptions in the fairness literature (see Bobocel, 2021), the MPA also provides an integrative structure to guide this important conversation.

To build our theoretical foundation, we begin by examining how people can use a broad range of information to inform their fairness perceptions beyond the well-established justice rules. Next, we identify the theoretical processes underlying how fairness perceptions can dynamically change as motives change. We then build from this dynamic perspective to outline how one's perspective (e.g., as recipient, actor, and observer) can influence this perceptual process as well as how fairness perceptions can be socially constructed and negotiated among parties. We conclude with theoretical and practical implications and an agenda to advance future research.^2^

## Examining Fairness as a Subjective and Motivated Phenomenon

Traditionally, the fairness literature has focused on people's assessments of justice rules (i.e., normative standards) to reflect their fairness perceptions (Rupp et al., 2017). Justice rules have been categorized into four dimensions (see Colquitt, 2001). Distributive justice focuses on the fairness of outcomes (e.g., whether outcomes reflect inputs), procedural justice focuses on the means used to derive the outcomes (e.g., whether the procedures were applied in a consistent manner), interpersonal justice reflects the fairness of interpersonal treatment (e.g., being treated with dignity and respect), and informational justice reflects the extent to which appropriate information was provided (e.g., whether an adequate explanation was provided). While scales assessing justice rules were never intended to directly assess “fairness” (see Colquitt, 2001), the literature often treats these rules and dimensions as being almost, if not completely, synonymous with subjective fairness perceptions (see Rupp et al., 2017).

However, emerging evidence challenges the notion that “what seems fair depends solely on what seems just” (i.e., that justice rules equate to fairness perceptions; Rodell et al., 2017, p. 14). For instance, there are significant concerns related to whether justice rules adequately reflect fairness perceptions; many of the rules were identified from scholars’ intuitions rather than empirical evidence and novel rules beyond the original rules have also been identified (e.g., Brown et al., 2010; Fortin et al., 2019). Moreover, the weights given to each rule can vary from person to person (e.g., German et al., 2016). There can also be differences in how people conceptualize what it means to be fair (e.g., managers may more narrowly define fairness and emphasize different rules than employees; Long, 2016). Further, fairness perceptions can reflect information that is unrelated to the justice rules, including affect (e.g., Barsky & Kaplan, 2007), characteristics of the person who is enacting the justice rules (e.g., charisma; Rodell et al., 2017), and observing others’ fairness reactions (e.g., Hillebrandt & Barclay, 2017), to name a few. Thus, fairness perceptions can incorporate diverse information from a multitude of sources.

Given that fairness perceptions are inherently subjective (i.e., people may draw on and differently weight information), this raises questions related to *what* information is used in fairness perceptions, *when* and *why* this information is emphasized, and *how* this can change over time. To answer these questions, we integrate the Brunswikian lens approach (e.g., Brunswik, 1952) with a motivated cognition perspective (e.g., Kunda, 1990) to identify the theoretical processes underlying the subjective, motivated, and dynamic nature of fairness perceptions.

## Marbles and Headlamps: Integrating the Brunswikian Lens Approach With a Motivated Cognition Perspective

Before outlining the MPA, we begin with an illustrative analogy that captures the key aspects of subjective and motivated perceptions. The streetlight effect is an allegory that tells the story of a person who has lost their keys in a park at night (see Freeman, 2010). However, instead of searching for their keys in the darkness of the park, they look for their keys under a nearby streetlamp—when asked why they would choose to search under the streetlamp instead of closer to where the keys are likely to be, they reply “because the light is better here” (Freeman, 2010). This allegory highlights how people focus their attention “where the light is better [rather] than where the truth is more likely to lie.”

Now imagine the same scenario but using a miner's headlamp instead of the streetlamp. People can look wherever they would like, but the headlamp's beam only illuminates where they choose to look. Imagine that a person is looking for information to inform their fairness perception. Think about the available information as marbles spilled onto the ground in the dark, with each marble representing a piece of information or “cue.” For example, the marbles may represent whether there was equity in how rewards were allocated, the manager displayed empathy, the information was delivered in a timely fashion, and so on. Individuals must decide where to shine their headlamp and which marbles to collect.

We argue that people's motives (headlamps) govern which marbles (cues) they see, what marbles they ignore, and when they turn the headlamp off (i.e., choose to stop looking). However, we also acknowledge that a person's search for marbles can be influenced by others. Imagine that the person believes that they have found enough marbles, but an observer tells the person that more marbles are just a little farther than where they are looking. This may motivate the person to expand the scope of their search. By contrast, another observer may tell the person that some of the “marbles” are in fact pebbles and that these should not be included in the marble bag. In these cases, interacting with others (and their motivated perceptions) can expand or alter one's marble search and/or impact whether the person even perceives something to be a marble.

Putting this analogy into theoretical terms, the Brunswikian lens approach emphasizes the importance of understanding that situations are made up of a multitude of cues (the marbles) that can be used (weighted) to form a perception (Brunswik, 1952) whereas a motivated cognition perspective explains why people are motivated to attend to and weight some marbles more than others (i.e., where they shine their headlamp). Below, we overview key tenets of the Brunswikian lens approach and a motivated cognition perspective. Our general argument is that integrating these approaches into the MPA can illuminate what cues are selected and weighted to arrive at a fairness perception and why this occurs.

### The Brunswikian Lens Approach: The “Marbles”

The Brunswikian lens approach is based on the notion that people collect and integrate information (i.e., cues) to inform their perceptions (Brunswik, 1952). That is, people can select and combine cues to reach their best estimates of reality (i.e., to create an accurate perception). However, there are a multitude of cues that are available in any situation and flexibility in how one weights these cues. Further, cues can be imperfect indicators of the situation, some may be more central whereas others may be more peripheral. The challenge is to determine which cues should be selected and how these cues should be weighted to “accurately” perceive the situation and effectively navigate one's environment.

The classical Brunswikian lens approach was developed in the context of visual perception and assumes that there is an objective reality to perceive, or best decision at which one can arrive. Indeed, some studies use an accuracy index to capture the degree of congruence between a perceiver's perception and reality (e.g., Schultheiss & Brunstein, 2002). Within the context of fairness, we argue that an objective reality does not exist; the best that can be hoped for is a shared subjective reality. As such, it is also critical to consider how *motives* can influence how people engage in cue choice and cue weighting to inform their fairness perceptions. To address this question, we now turn to the headlamps (i.e., a motivated cognition perspective).

### A Motivated Cognition Perspective: The “Headlamps”

The essence of a motivated cognition perspective is that people are active and motivated processors of information (Kunda, 1990). Importantly, people's motives can influence how they select, evaluate, and weigh the information that ultimately shapes their perceptions (see Table 1 for examples). From this perspective, motives are defined as “any wish, desire, or preference that concerns the outcome of a given reasoning task” (p. 480). The motivated cognition literature has identified two main categories of motives: nondirectional and directional (e.g., Kruglanski, 1996; Kunda, 1990). Nondirectional motives (e.g., accuracy and closure) can influence how information is processed but this processing occurs without consideration for the conclusion that the processing may lead to (e.g., Kunda, 1990). For example, accuracy motives can focus people on selecting and weighting cues to obtain the most accurate conclusion possible, regardless of what that conclusion is (Kruglanski & Webster, 1996). By contrast, directional motives (e.g., instrumental and relational) guide the selection and use of cues toward a desired conclusion.

**Table 1.** Examples of Nondirectional/Directional Motives and Effects on Fairness Perceptions.

Motive type	Motive	Goal “why”	Cue search characteristics “what”	Cue weighting “how”	Potential consequences
**Nondirectional**					
	**Accuracy**	Arrive at a perception that best reflects an unbiased (“objective”) assessment	Search for and attend to all relevant cues	Actively try to suppress cognitive biases and evaluate cues in an impartial way Attempt to optimally weight available cues to represent an unbiased evaluation	Not always sustainable/possible: heavily dependent on time, availability of information and availability of cognitive resources If all parties are motivated by accuracy, there is a higher likelihood that parties will have common or shared perceptions
	**Closure**	Arrive at a perception that allows the perceiver to come to a conclusion that will enable them to move forward	Search for and attend to cues until enough information to reach a conclusion is gathered	Cues that enable a conclusion are more heavily weighted, even if they are not the most relevant to the situation	Perception may not include or appropriately weight relevant cues Parties may move on but may not have shared perceptions or common cue sets
**Directional (related to why people care about fairness)**					
	**Instrumental**	Maximize personal outcomes (e.g., economic outcomes)	Attend to cues that signal whether one has control over outcomes, future outcomes, and how one is treated relative to others	Cues related to the valence of outcomes more heavily weighted	Heightened sensitivity to under compensation for oneself and diminished sensitivity to over compensation for oneself May prioritize one's own interests over the interests of and/or justice for others
	**Relational (including self-enhancement, identity maintenance)**	Fulfil need to feel a valued member of a group (positive self-regard, status) and have a sense of belonging	Search for cues relevant to how others see oneself and one's standing in the group or the standing of the group itself	May weight information from in-group versus out-group members more heavily	May perceive advantaging one's own group members or disadvantaging non-group members as fair May selectively value fairness for close “circles of justice”
	**Moral**	Uphold moral rules and the importance of moral duties (e.g., “oughts”)	Search for cues related to moral standards/violations and moral mandates	Cues that relate to individual's moral standards heavily weighted	Value fairness for its own sake and because it is the “right thing to do,” even if it does not benefit the self May be closed to including any cues that contradict own moral standards, making it impossible to reach shared perceptions in case of diverging moral standards
**Directional (related to upholding beliefs about fairness)**					
	**Justice motive**	Uphold belief that the world is a fair place (i.e., people get what they deserve)	Search for cues that can maintain belief (e.g., search for cues that treatment for oneself or others was deserved and therefore fair)	Cues that allow maintenance of a belief in a just world heavily weighted	If unable to restore justice through action, then may persuade themselves that negative treatment or outcome is justified (e.g., being unable to alleviate the perceived suffering of a target can lead to victim derogation)
	**System justification**	Uphold belief that the existing social order in which one is embedded is justified and fair	Search for cues that can enable people to support the extant system	Cues that can be used to justify the status quo are heavily weighted	May perpetuate and justify specific unfair events to maintain the belief that the system itself is fair, even if this means justifying unfairness that one experiences

This suggests that the same person may be able to justify different conclusions when different motives are activated (Kunda, 1990). However, while people may employ directional strategies to facilitate their preferred conclusion, they are not free to arrive at any judgment they wish—even when motivated to do so. Directional motives are constrained by an “illusion of objectivity” (Kunda, 1990, p. 483), such that people can believe what they want but only to the extent that reason, cognitive capacity, and the available information permits. That is, people are typically constrained by whether they can provide a seemingly rational justification for their preferred conclusion (Pyszczynski & Greenberg, 1987).

The fairness literature has focused on three categories of directional motives that reflect why people care about fairness (Cropanzano et al., 2001). Instrumental motives (e.g., economic self-interest) focus on having control over one's outcomes and receiving beneficial outcomes, relational motives (e.g., positive self-regard and belonging) focus on how fairness can fulfill one's social and relational needs, whereas moral motives focus on providing meaning by upholding moral duties or norms. Although these motives have typically been used to reflect why people care about fairness, there is increasing recognition that these motives can also shape fairness perceptions (for discussions, see Barclay et al., 2017; Blader & Bobocel, 2005).^3^

In the next section, we develop the MPA by outlining how people are motivated to select and weigh information, how multiple motives may be activated, how motives can work together or in opposition to shape fairness perceptions, and how shifting motives can dynamically influence one's fairness perceptions.

## The Dynamic Nature of Fairness Perceptions: Understanding the Motivated Perceptual Processes of the Perceiver

**Figure 1.**
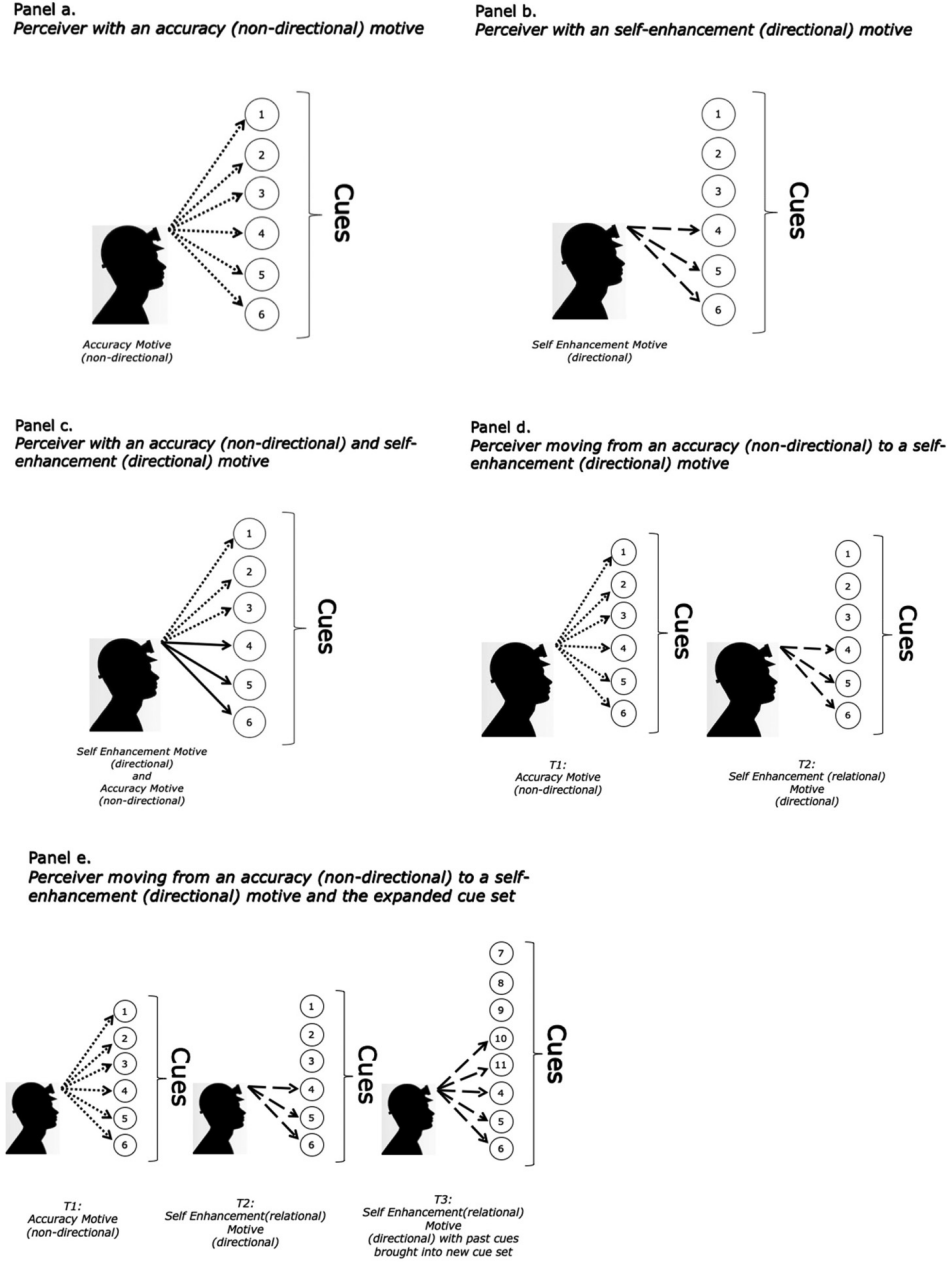
Illustrative example of how motives can impact cue selection for a single perceiver.

A key tenet of the MPA is that fairness perceptions are a function of the available cue set, the perceiver's motives, and the dynamic interaction between cue sets and motives. To illustrate, let's assume that a perceiver has a clean slate when assessing fairness (i.e., does not bring any cues from the past but starts with a new set of cues/marbles). The perceiver has immediate access to several cues (e.g., what is said and what is received). Yet, the perceiver's motives can influence the cues that are attended to and how these cues are weighted to inform fairness perceptions. Consider the case of a perceiver who has a nondirectional motive for accuracy (see Figure 1, panel a). An accuracy motive can prompt the perceiver to engage in an intensive search for cues (i.e., the perceiver attends to all available cues; cues 1–6 in Figure 1) and assign weights that reflects the perceiver's assessment of each cue's importance. This information is then used to arrive at a fairness perception. Now consider the same perceiver but instead of a nondirectional accuracy motive, the perceiver has a self-enhancement motive (i.e., a directional motive to arrive at a fairness perception that feels good; see Figure 1, panel b). This motive changes the relevant cues. Instead of considering all possible cues, those cues that have a self-enhancing element (cues 4, 5, and 6) are selectively attended to and preferentially weighted. Finally, consider this same perceiver yet again, except in this case the perceiver has two motives activated simultaneously—a nondirectional motive for accuracy *and* a directional self-enhancement motive (see Figure 1, panel c). This perceiver is motivated to hold an accurate fairness perception, but also wants to feel good about themselves. In this case, while all cues may be considered, those cues that have a self-enhancing element are more heavily weighted (as indicated by the solid line). This suggests that the interplay between motives depends on the overall desired perception. Notably, across these situations, the same cues are available to the perceiver. However, one's motives can guide the way that the cues are selected, evaluated, and weighted, which can ultimately result in vastly different perceptions based on the same set of available cues.

Importantly, perceptions (and the cue sets and motives driving them) are not static. Instead, cues, motives, and therefore fairness perceptions can change as the perceiver's experiences and expectations change. Returning to Figure 1, a perceiver who recently joined an organization may initially operate from a nondirectional accuracy motive and actively search for cues as they attempt to arrive at an accurate perception about their organization and the treatment they can expect (see Figure 1 panel d, T1). Imagine the perception arrived at is that the organization is unfair. As time passes, the perceiver may no longer be motivated to accurately perceive the fairness of the organization (i.e., hold a nondirectional motive). Instead, the perceiver may adopt a self-enhancing motive that shapes the way that cues are selected and weighted.^4^ As depicted in Figure 1 (panel d, T2), rather than attending to the entire set relevant to the organization (cues 1–6), the perceiver may selectively attend to cues associated with the current motive of self-enhancement (cues 4–6). In doing so, they may not only weight the cues associated with the motive more strongly, but may also discount (i.e., reduce the cue set) or reweight cues that do not align with this motive, especially when these cues detract from their ability to perceive the situation in the desired manner. As this process unfolds over time, both the motives *and* the cue set can change. For example, past cues from other situations may be brought forward, expanding the cue set and shaping one's fairness perceptions (Figure 1, panel e, T3; cues 4, 5, and 6 are carried forward to fill out the cue set driven by the self-enhancement motive). Alternatively, the perceiver may seek additional cues to support the desired conclusion. However, this motivated cue selection and cue set expansion remains at the mercy of the illusion of objectivity. If, over time, the perceiver is confronted with repeated cues indicating that their perception does not align with the “reality” of the situation, then the perceiver may have to include and/or weight some of these cues (perhaps begrudgingly) in the face of overwhelming evidence, thereby shifting the fairness perception.

## Understanding the Perceiver in a Social Context: The Interplay Between Parties to a Fairness Interaction

As complex as the above process may sound, recognizing that the motivated perceptual processes underlying fairness perceptions can be distilled to cue sets and motives provides a foundation to understand not only how fairness perceptions emerge but also why parties to an interaction often hold dissimilar fairness perceptions.

### Basic Tenets Underlying the Interplay Between Recipients and Actors

**Figure 2.**
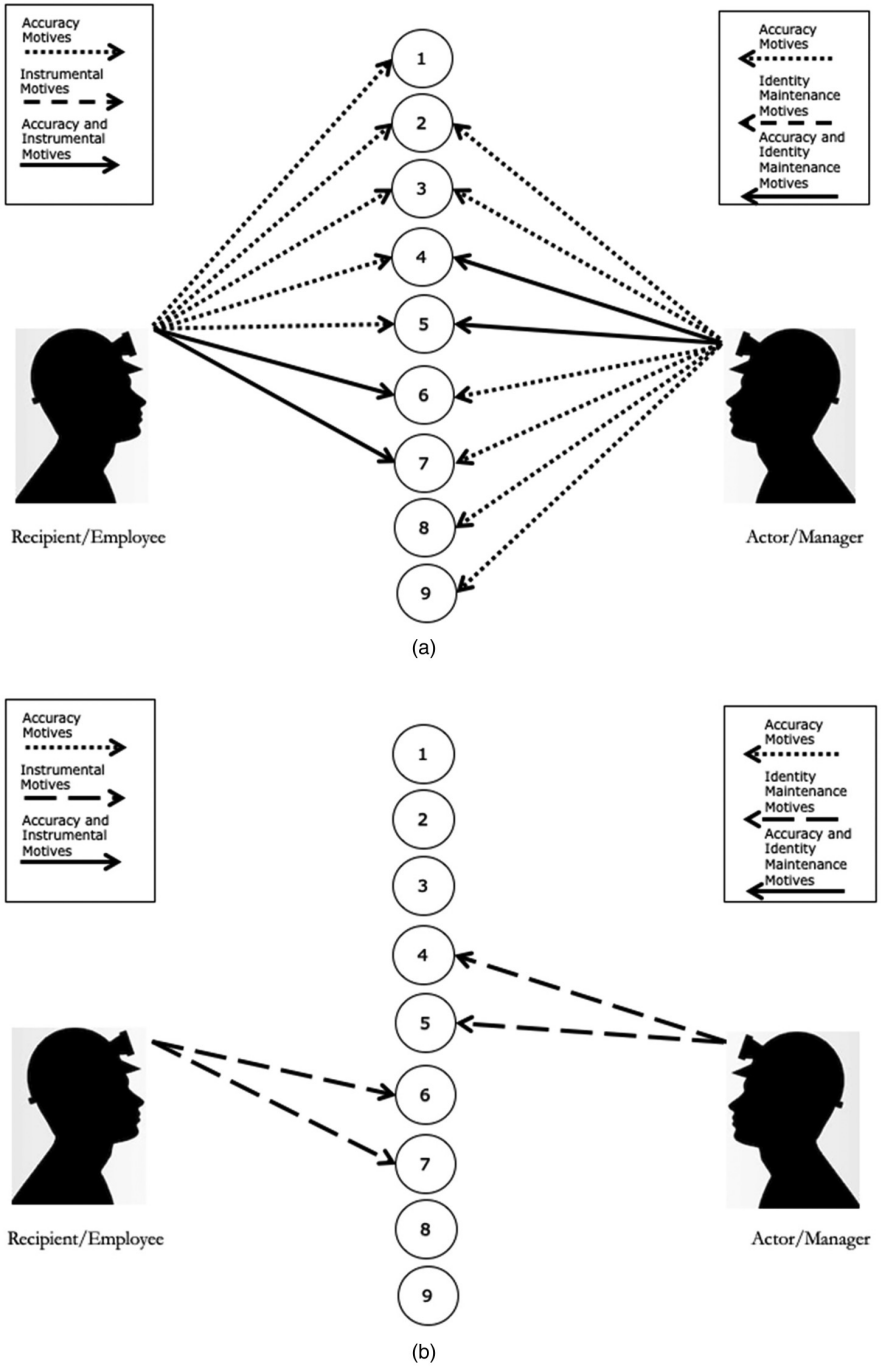
Examples of different cue sets and weightings between perceivers. (a) Illustrative example of resolvable predicament of unfairness. (b) Illustrative example of intractable predicament of unfairness.

Using a recipient-actor dyad, Figure 2a outlines how perceptual incongruency can emerge between parties based on the differing availability of cues alone. In this case, both parties have an accuracy motive and are expected to expend effort in a search for cues. While the cue sets for both parties share some overlap (cues 2–7), there are some cues that are available to the actor but not the recipient (cues 8 and 9) and vice versa (cue 1). Thus, even if parties are motivated to perceive the entire cue set, their differential access to the cues can impede their ability to do so.

Beyond the availability of cues, each party may differentially select, weight, and/or interpret the available cues. Consider a detailed explanation provided by a manager. The manager and employee may imbue the “same” cue with disparate meaning or differentially weight the cue. For instance, the manager may interpret the provision of an explanation in a positive manner (e.g., as a reflection of fairness since this adheres to an informational justice rule) whereas the employee may interpret the same cue in a negative manner (e.g., the manager is being patronizing). Each party may also experience disparate motives due to their roles. For example, an employee may have instrumental motives and more strongly weight cues related to the valence of the outcome, whereas a manager may be guided by identity maintenance motives to be seen as a fair manager and place more weight on cues that relate to the adherence to justice rules (see Figure 2b). Taken together, cue accessibility, cue interpretations, and the activation of disparate motives may create incongruency between the parties’ fairness perceptions.

Importantly, when actors and recipients experience discrepancies in their perceptions, they can experience a “predicament of unfairness” (i.e., the actor may perceive that they are acting fairly whereas the recipient does not; Bies, 1987; Cooper & Scandura, 2012; Whiteside & Barclay, 2015). However, the MPA suggests that some predicaments of unfairness are less intractable than others. For example, in Figure 2a, there is some overlap in the cue sets, with both parties being able to “see” the other's relevant cues. The presence of an accuracy motive also makes it more likely that both parties will weight those cues (i.e., use this information to arrive at a fairness perception). By contrast, in Figure 2b, both parties have directional motives that prompt them to attend to and weight very different cues. There may also be some situations where the parties do not share access to the same cues or where neither party holds an accuracy motive (i.e., neither party is motivated to perceive the broader cue set). Predicaments of unfairness will be more intractable when there is a lack of common perceptual cues.

When such perceptual discrepancies exist between the parties, the cues and motives in the situation may provide insight for how to resolve the predicament. For example, it may be helpful to ensure that both parties have access to the same cues and the same interpretation of these cues (e.g., enhance communication/sharing of information between the parties), consider how directional motives may be influencing the situation (e.g., engage in perspective-taking to determine whether the weighting of some cues may need to be reconsidered), and/or motivate at least one of the parties to hold an accuracy motive to help identify and resolve incongruencies.

While both parties may benefit from the above strategies, managers may be particularly well-suited to implement these strategies, due to the legitimate power vested in their role. For example, managers can share their cue set with those impacted by their decisions (e.g., Chun et al., 2018) or engage in “sensegiving” by providing others with guidance and interpretations that can influence the motives of the other perceivers and/or focus other perceivers on which cues to select, attend to, and weight (e.g., Maitlis & Lawrence, 2007). Managers may also be especially likely to contribute novel cues to the situation through their behaviors. For example, Blader and Chen (2012) demonstrated that when managers are other-oriented, they adhere to procedural justice rules more than when they are interested in power. Finally, cues from others can prompt managers to change their behaviors. For example, if managers receive cues from employees that their behavior was unfair, they may be motivated to expand the cues in the situation in ways that can enable them to maintain a favorable social identity (Whiteside & Barclay, 2015).

### Understanding the Interplay Between Actors and Recipients as a Dynamic Process

Differences in cues and motives may also influence how the fairness situation unfolds and dynamically shifts over time. For example, the parties may activate and/or react to the other's motives, which may impact the cue set that is available to and/or used by both parties. Consider an employee who questions the fairness of the manager's behaviors—this may prompt the manager to provide new cues to the employee (e.g., by explaining the procedures more thoroughly) and/or activate identity motives in the manager that can impact the manager's behavior (e.g., changing subsequent behavior to appear fair, Oç et al., 2015, 2019). The manager's behaviors may also impact the recipient's motives and perceptions, with the reaction of the employee then serving as a cue for the manager. Thus, the parties may dynamically influence each other (e.g., by altering cue sets and activating motives in each other).

However, this begs the question of whether managers should only focus on cues that are aligned with the recipient's current motives. For example, an employee with supposedly instrumental motives may respond well to cues that relate to their personal benefit during the event. Yet, the employee may be prompted by new information to change their motives and/or to reassess their fairness perception at a later time (e.g., when they are discussing the event with a colleague). Furthermore, individuals have limited self-insight into the relative weighting of different fairness norms they use, which suggests that they may also not be able to communicate the relative importance of the different motives they hold at any point in time (German et al., 2016; Rousseau & Aquino, 1993). Therefore, the manager may be well-served to provide cues that align with a range of motives (e.g., instrumental, relational, and moral).

Motives may also shift during the exchange. For example, while each party may enter the interaction with certain motives activated, these motives may change in response to the other party or one's own needs. Consider a manager who enters the situation with a closure motive but the employee raises questions that imply that the manager is being unfair. This may prompt the manager to shift to an identity maintenance motive and highlight the fairness of their behaviors. Alternatively, an employee may have a relational motive at the start of a meeting but an accuracy motive is activated when the employee realizes that they are receiving bad news and it is important to have a complete understanding of the situation. As the employee attempts to expand their cue set (e.g., by asking questions of the manager), this may also activate motives in the manager (e.g., relational motives to demonstrate care and concern to the employee). Thus, there may be interplay between the actor and recipient that may prompt motives to shift and/or multiple motives to be activated at once.

Further, cues for an event may not be limited to the event itself but may also come before (e.g., when a manager prepares to deliver bad news) or after the event (e.g., when a manager tries to help an employee transition from the news after it has been delivered; see Bies, 2013). Cues that are salient at earlier stages of the interaction may also shape subsequent cues and motives. For example, cues and motives that are active during an event may prompt the individual to continue to seek information afterwards to reduce the ambiguity in these cues and/or to add new cues (e.g., the procedure was not described clearly when the decision was communicated, so the employee invests time searching for procedural cues after the event has occurred). Given that the parties may have differential access to cues in the various stages, the parties may also add to the cue set for others by expanding the temporal scope for the interaction (e.g., a manager may provide information about the preparation or transition stages to expand the recipient's cue set).

### Understanding the Interplay Between Actors, Recipients, and Third Parties

Similar to the interplay between recipients and actors, third parties may also influence fairness perceptions by altering the cue set, the weighting of the cues, and/or by shifting motives. For example, peers can influence what cues recipients attend to (e.g., Jones & Skarlicki, 2005) or help recipients make sense of an event (e.g., Degoey, 2000; Lamertz, 2002). Further, third parties may also help the recipient interpret and assess the relevancy or credibility of cues (e.g., whether others think that the manager is sincere; Lamertz, 2002) or provide their own experiences as a cue (e.g., Hillebrandt & Barclay, 2017), especially when there are multiple recipients who have shared experiences with the same actor (Lind et al., 1998). Similarly, actors may be especially motivated to ensure that their decisions are defensible when third parties may evaluate these decisions (e.g., Kunda, 1990). This suggests that all parties (i.e., actors, recipients, and third parties) may dynamically influence and be influenced by fairness perceptions, although there may be moderators for these effects (e.g., relationship closeness).

## The Importance of Perspectives

As suggested by the above discussion, fairness is an inherently social phenomenon that “transpires in a social context involving multiple parties” including recipients, actors, and potentially third parties (Brockner et al., 2015, p. 104).^5^ While the MPA indicates that all parties to a fairness situation operate from the same basic perceptual processes (i.e., using cue sets and motives), it also recognizes that one's perspective (e.g., role) can be influential in determining what motives are triggered as well as what cues are accessible and perceived. In this section, we consider a real-life example to demonstrate this point, followed by a theoretical discussion of how perspective can impact motives and cue sets.

### The Case of Kerviel: A Demonstration of the Influence of Disparate Perspectives

In 2010, Jérôme Kerviel, a trader for Société Générale (a French multinational banking and financial services company), was convicted for his role in the loss of 4.9 billion Euros, which nearly bankrupted Société Générale. While Kerviel's conviction was lauded by those who considered his actions to be a breach of trust, Kerviel characterized it as a “major injustice.” To justify his perspective, Kerviel emphasized that he was a “small cog in the machine” that was pressured by his supervisors to make “more and more” (Pauly, 2010). In 2016, an appeals court agreed with Kerviel and overturned the initial ruling, noting that deficiencies in the bank's risk management and security systems led to the massive losses. A subsequent tribunal in the French court system dedicated to labor law went further and ruled that Société Générale had to pay wrongful dismissal charges: “Société Générale can’t pretend it was not aware of Kerviel's fake operations” (Samuel, 2016). Société Générale's representatives described this decision as “scandalous” and “counter to the facts” (Samuel, 2016). As for Kerviel, he became a folk hero in France, where many see him as a scapegoat in a corrupt capitalist system, and where his story has inspired T-shirts, a song, and even a movie (National, 2016).

This example highlights how fairness perceptions can be shaped by motives, cues, and perspective. Each party—Kerviel, Société Générale, and the courts—are motivated to be seen as fair and/or justified. Kerviel, aside from his financial interest in being judged not guilty, is motivated to position himself as a victim of the system and counter the narratives offered by the others (e.g., prosecuters). From Kerviel's perspective, a fair judgment would focus on the context in which he was operating. By contrast, Société Générale is motivated to see the financial fiasco as a result of a bad apple rather than a bad barrel, especially given the large media interest in the case. If the losses were part of a systemic problem, then the reputational threat would be much greater. For them, a judgment that focuses on Kerviel as a rogue trader is the one that they deem fair. Finally, from the perspective of the French court system, the judges are motivated to thoroughly consider all available evidence and come to as accurate a judgment as possible to be seen as fair, especially with the case receiving massive public attention. These disparate motives can shape where each party focuses attention and what information is deemed relevant for their fairness perceptions. Moreover, each party is influenced by the presence of others that they deem relevant, including the courts for Kerviel, investors and customers for Société Générale, and the general public for the courts. Thus, these social and contextual factors can shape the motives that are activated, the cues that are attended to, and how the discourse unfolds.

### The Perceiver's Perspective and Motives

As illustrated in the example above, motives can be activated by one's role. For instance, in terms of directional motives, moral motives have traditionally been associated with third-party observers because they are not directly impacted by the situation (e.g., Folger, 2001). However, recent research illustrates the impact of moral motives for actors, who may engage in moral disengagement to relieve their concerns or even leave their role if they feel obliged to engage in unfair actions (Zwank et al., 2022).

Nondirectional motives may also be differentially activated depending on one's role. For example, accuracy motives may be critical for recipients who wish to assess the risk for future exploitation (e.g., Lind, 2001), for parties who must justify their fairness judgments to others (e.g., human resource managers, and lawyers), and/or those who are assigned a role that prescribes accuracy (e.g., arbiters and judges). Closure motives may also be impactful, especially when parties do not have the cognitive capacity or time to process an event (e.g., their attention is needed elsewhere) or when the event fits their pre-existing heuristics (e.g., Lind, 2001). In these instances, directional and nondirectional motives may interact (e.g., attempting to enact justice in a time constrained situation while maintaining a positive self-image, e.g., Camps et al., 2019).

Finally, contextual factors may also activate a given motive differently depending on one's role. For example, nondirectional motives such as instrumental (e.g., union members protesting mistreatment of others may also be motivated to prevent future mistreatment for themselves, Skarlicki & Kulik, 2004) or relational motives in third parties may be particularly relevant when third parties identify with or belong to the same group as the recipient (e.g., the persistent injustice effect; Davidson & Friedman, 1998). Empirical evidence has shown that layoff survivors can have strong reactions to unfairness, especially when survivors strongly identify with the layoff victims and perceive layoff victims to be within their “scope of justice” (e.g., Brockner, 1990). Third parties may also be prone to a just world motive—a directional motive that may prompt the selection and weighting of cues that maintains one's belief that the world is a fair place (Lerner, 1980).

### The Perceiver's Perspective, Motives and Cue Sets/Weighting of Cues

Beyond the differential activation of motives, we argue that perceivers may differentially weight cues and/or have access to different cues based on their perspective. Indeed, even when parties have the *same* motive activated, they may differentially select or weight cues. Consider moral motives. Managers may be sensitized to the importance of fulfilling the moral obligations and responsibilities inherent to their formal managerial role. Whereas this may focus managers on moral cues about their own *behaviors*, recipients may focus on the morality of the manager *as a person*, while third parties may consider cue sets related to the behaviors, the manager, *and* contextual cues. For example, there is evidence that recipients may consider attributes of the actor as part of the relevant cue set (e.g., charisma; Rodell et al., 2017). By contrast, actors may not be aware of these attributes or may not perceive these cues to be relevant. The actor-observer effect also demonstrates that actors are more likely to weight dispositional information when evaluating others, but more strongly weight situational cues when evaluating themselves (Jones & Nisbett, 1971). Consider self-enhancement motives as another example. This motive may prompt recipients to focus on positive attributes associated with themselves (e.g., their own deservingness) or cues that can contribute to feelings of self-worth, confidence, and competence (Weiner et al., 1979). By contrast, managers may focus on their own positive attributes (e.g., time and effort devoted to “being fair”; adherence to justice rules) or cues that contribute to their reputation, standing, or own identity to maintain a belief that they acted in a fair manner (e.g., Camps et al., 2019).

Depending on the motive, the perceiver may also bring in old cues into new situations and/or their expectations may impact the weighting of cues. For example, recipients’ past experiences with a manager may provide cues that can impact current fairness perceptions (e.g., Rubenstein et al., 2019) whereas managers may recall similar interactions with other employees. However, others may not have access to these cues, perceive them as less relevant, and/or fail to recognize differences related to the disparate perspectives or the qualitative differences in power dynamics embedded in the roles. Similarly, recipients may be less likely to notice the efforts of others (e.g., Burrus & Mattern, 2010)—especially when these efforts did not result in outcomes—and therefore less likely to recognize the efforts that actors made toward enacting justice (e.g., underweight cues related to the manager's adherence to justice rules). Recipients may also underestimate what level of reward is fair for others but overestimate what level of reward is fair for them, thereby impacting cue selection/weighting.

Third parties may have a more limited and/or disparate cue set than recipients and actors because they are less immersed in the situation (e.g., rather than first-hand knowledge, they may witness only parts of an event or learn about the situation through other means, such as rumors). However, third parties may have access to other cues that may not be available to other parties (e.g., contextual factors), may be less influenced by some cues (e.g., the favorability of the outcome), and/or may be influenced by biases related to their relationships with the recipient or the actor (e.g., prior negative experiences with an unfair actor or their own personal experience of injustice; Kray & Lind, 2002). For instance, third parties may not always experience congruent emotional reactions (e.g., empathy) to recipient's outcomes. Instead, the presence of incongruent emotional cues (e.g., schadenfreude) may lead third parties to perceive objectively unfair disadvantages as fair (e.g., under conditions of low liking or high psychological distance; Blader et al., 2013). Moreover, third parties may use different strategies than recipients or actors to determine how to select, attend to, and weight the available cues. For instance, recipients and actors may perceive cues originating from their own perspective as valid but question the validity of cues provided by the other party. By contrast, third parties may assess the validity of cues originating from both sources before weighting the cues. Taken together, the available cue set and how it is evaluated or weighted can differ across parties, even when the same motive is activated. This can create the possibility that parties need to negotiate or socially construct fairness perceptions. We explore this below.

## The Motivated Social Construction of Fairness Perceptions

Implicit in our theorizing has been the notion that fairness perceptions are socially constructed—while each party can perceive an interaction with their own subjective lens, parties may try to persuade or negotiate with others to create a shared subjective reality by enhancing each other's cue set, encouraging the reweighting of cues, and/or activating different motives. By highlighting these dialectic influences, the MPA recognizes that there are multiple parties to a fairness interaction who may hold disparate perceptions and these parties may try to reconcile differing points of view through implicit or explicit discourse. That is, people can socially construct and negotiate fairness perceptions through interactions with motivated others.

**Figure 3.**
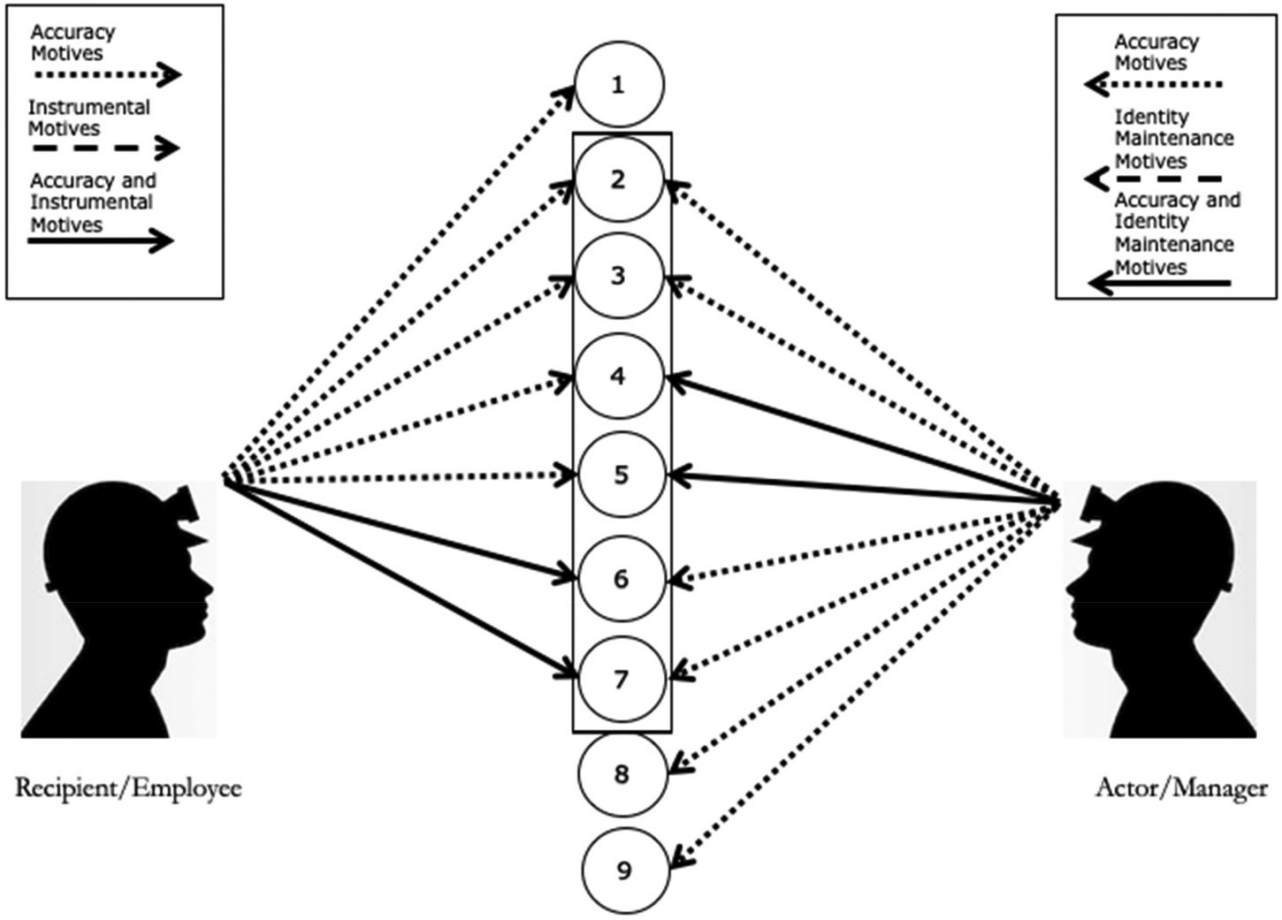
Managing predicaments of unfairness: the zone of acceptance.

Dialectic processes are fundamentally an attempt of one party to persuade another party to share a cue set and/or weight cues in a similar manner. However, the ease of this task may depend on the degree overlap between the cue sets. Building on the predicament of unfairness discussed earlier, we argue that the overlapping cues may be a starting point for the dialectic by creating a “zone of acceptance” that is shared by both parties (see Figure 3, cue set within the rectangle). The dialectic processes relate to how the parties expand or contract this “zone of acceptance” over repeated interactions. For example, a manager may recognize that the employee is focused on a different set of cues and persuade the employee to use similar cues as the manager to create a shared zone of acceptance. Alternatively, the manager may make additional cues salient by encouraging the employee to read documentation or consult with human resources, which may activate an accuracy motive by making the recipient feel that they need to justify their perceptions. Further, the manager may trigger other motives that can shape the interaction. For example, the manager may limit the time available for the dialectic to unfold to trigger a closure motive. Alternatively, the manager may use an apology to trigger a relational motive in the recipient and add new cues to the cue set (i.e., the acknowledgement of responsibility). Regardless of which motives are activated, once a fairness perception is created and/or an action taken, these perceptions and behaviors can become part of the cue set that is available in subsequent rounds of the dialectic and trigger subsequent motives.

Importantly, dialectic processes are discourses that can be shaped by multiple parties—these interactions do not have to occur simultaneously but rather tend to unfold over time. For example, interactions between some parties (e.g., actor-recipient) may lead to interactions with other parties (e.g., recipient-third party) that can initiate, continue, alter, or close the discourse. These discourses may also expand or reduce the number of parties that are involved. An employee may socially construct a fairness perception during an interaction with their manager, reshape this perception by talking with a colleague, and then further negotiate the fairness perception when they return to talk to the manager about their concerns. Taken together, dialectic processes emphasize the temporal and dynamic nature of fairness perceptions, which evolve over time and through interactions with motivated others.

## General Discussion

The MPA highlights the advances that can be made by grounding theorizing on the inherently subjective and motivated nature of fairness perceptions. These advances include outlining the dynamic and dialectic processes that have been previously obscured by a narrowed focus on static and objective conceptualizations of fairness. By delineating these processes, the MPA provides a foundation for unifying and enhancing our theoretical understanding of fairness as well as providing insights into how fairness issues can be effectively managed in the workplace. Below, we outline key theoretical insights provided by the MPA, opportunities for future research, and practical implications.

### Embracing the Subjective and Motivated Nature of Fairness Perceptions

By embracing the fundamental assumptions that fairness is inherently subjective and motivated, the MPA explains why people can hold dissimilar fairness perceptions, even when the underlying “facts” of the situation are the same. Rather than imposing a static and objective conceptualization of fairness on perceivers or restricting fairness perceptions to a narrow set of rules (see Rupp et al., 2017), the MPA broadens our understanding of how people perceive fairness. Given that perceptions form the perceiver's reality, capturing the subjective nature of perceptions is critical to understand their effects and how they can be effectively managed.

By grounding on the assumption that fairness perceptions are motivated, the MPA also creates the opportunity to examine *what*, *why*, *when*, and *how* motives can guide the perceptual process (e.g., the selection, weighting, and interpretation of cues) and raises important new questions. In Table 1, we overview examples of the motives that are especially important for the formation and maintenance of fairness perceptions. However, it may also be helpful for future research to develop a typology of the motives (including directional and nondirectional) that are influential for fairness. While the motivated cognition and fairness literatures have identified some motives, a more comprehensive approach focusing on the impact of various motives on fairness perceptions and their interplay is likely to yield fruitful insights.

Exploring how motives can impact the durability/malleability of fairness perceptions may also be insightful. For example, fairness perceptions that emerge from a nondirectional accuracy motive may be more durable because they include a broader cue set and involve the deliberate processing of the cues. While many directional motives may result in more malleable perceptions (e.g., it may be easier to expand rather than shrink a cue set), there may be a subset of directional motives (e.g., those that are emotion or value-laden) that result in durable perceptions (e.g., Mullen & Skitka, 2006). This highlights the importance of examining how motives impact information processing (e.g., automatic vs. controlled processing; see Barclay et al., 2017).

The MPA also provides a unified foundation that identifies the underlying perceptual processes common to all perceivers while also recognizing distinctions between perceivers and how these distinctions can shape this underlying process. This includes how one's role (e.g., manager, employee, and third party) can impact perceptual processes by influencing the content (e.g., cue availability), motives (e.g., what motives emerge and how motives are experienced), and/or emergence of perceptual biases. This can provide insights into why discrepancies are likely to emerge and how these discrepancies can be reconciled. For instance, discrepancies in cue sets may be resolved by engaging in perspective-taking or by using communication strategies that can make cues available to the other parties. By contrast, when both parties have access to the same cues but are focused on different cues (e.g., have different motives activated), it may be more effective to activate a motive in both parties that will encourage overlap in the cues (e.g., an accuracy motive) and/or shift people away from motives that may reduce their search for additional cues (e.g., closure motive). Thus, the MPA sheds light on the underlying motivated perceptual process while integrating how perceivers’ roles may shape this process. These insights are critical for understanding and managing fairness perceptions between the parties.

### The Dynamic Nature of Event and Entity Fairness Perceptions

We focused our theorizing on fairness perceptions in a general sense and did not distinguish between event-based (i.e., evaluations related to a specific situation) or entity-based perceptions (i.e., evaluations of a person that transcend specific situations; see Cropanzano et al., 2001). We made this choice because the MPA can be applied to the processes related to both event and entity fairness perceptions. However, the nature of the cues used may shift depending on whether one is assessing the fairness of an event or a person. For example, event fairness perceptions may emphasize details of the events (i.e., what was said) whereas entity fairness perceptions may be more likely to include details that transcend events (e.g., attributes of the actor). Several theoretical implications for event and entity perceptions emerge from the MPA.

First, fairness perceptions can evolve as an event unfolds. Consider a promotion context. Employees’ fairness perceptions may shift from when they submit an application for promotion, while they wait to hear the decision, during the meeting with their manager, and as they deal with the transition after receiving the decision (see Bies, 2013). This shifting may continue after the event is ostensibly over, such as when the perceiver later reflects on the event or reinterprets it based on a broader set of events. For example, an employee may initially perceive a promotion denial as unfair but may later recognize other cues that change this perception (e.g., perceive that their manager was looking out for them since the promotion was different than expected). This shift in fairness perceptions may be due to the different motives that can be activated across these stages, the emergence of new cues, and/or through interactions with others (e.g., managers, colleagues, human resources, and significant others) that can change the way that people select and weigh cues depending on what motive(s) are activated at any given time. Moreover, this suggests that it may be informative to examine how cues influence initial fairness perceptions as well as how these cues and processes may inform how people remember and reinterpret the situation.

Given the temporal unfolding of events (i.e., preparation, delivery, and transition stages), this also raises the question of what should be considered an “event.” We propose that it may be informative to move away from narrow and static conceptualizations of events (i.e., an emphasis on the delivery stage) and toward a broader context that includes all the stages related to a precipitating event (see Bies, 2013; Kitz et al., 2023), including how a precipitating event can be connected to other events and/or situations over time (e.g., the denial of a promotion may provide marbles that are considered when an employee subsequently fails to also receive an adequate raise; also see Morgeson et al., 2015). Thus, we propose that examining “fairness episodes” may be a fruitful way to consider how events unfold over time and the processes underlying these temporal dynamics (see also Hillebrandt & Barclay, 2013).

Second, the MPA highlights that within-person variation can occur with fairness perceptions related to events *and* entities while also providing the theoretical basis for delving into the interplay between event and entity fairness perceptions. For example, while pre-existing entity perceptions have been shown to shape event perceptions (e.g., Choi, 2008), the MPA provides insight into the underlying processes (i.e., pre-existing entity perceptions can provide cues for interpreting events). Similarly, scholars have proposed that experiencing new fairness-related events can shift entity fairness perceptions (e.g., Jones & Skarlicki, 2013). The MPA provides an enhanced theoretical understanding of “why” this occurs by highlighting how events can provide new cues that can be incorporated into entity perceptions, while also recognizing that one's motives can impact whether these new cues are attended to.

Importantly, the MPA indicates that entity fairness perceptions can change, even in the absence of a new event. For example, within-person variation in event fairness perceptions (e.g., the expansion or reweighting of cues and/or changes in motives) can expand or alter the perceiver's entity-based cue set, how this cue set is used, and/or the motives that are activated. The MPA also provides a strong theoretical foundation for explicating why and how event and entity perceptions inform each other (e.g., by expanding the cue set, shifting the weights of cues, and/or changing one's motives). These insights are especially critical for managers. While it is often assumed that fairness perceptions may only need to be managed in the presence of major events, the MPA suggests that managing fairness perceptions is an ongoing process and that theorizing related to reactions to (un)fair situations may need to recognize this temporality.

Further, the MPA can provide insights into how people form entity perceptions through the aggregation of events (see Rupp & Paddock, 2010). Similar to the above discussion about how nondirectional and directional motives may influence cue selection for events, these processes may also extend to how people can differentially weigh various events when forming aggregate fairness perceptions. For example, a person who is motivated to have an accurate perception of their manager may try to carefully consider and weight each event when creating an aggregate entity perception, whereas a person who is directionally motivated may place more weight on events that communicated information aligned with this motive. Thus, the MPA highlights that motives (not simply new events) may shift entity fairness perceptions by influencing the way that people compile these events to form aggregate fairness perceptions. This insight is important because it highlights that predicaments of unfairness can impact entity perceptions, especially when people differentially weight events. For example, an employee may strongly weight an event in their entity perception of the manager, but the manager may not even realize that this event was considered unfair by the employee and/or recognize the importance of this single event, especially in light of other fairness events.

Finally, the MPA implies that the distinction between event and entity perceptions may be too rigid; event perceptions may leverage cues that are typically associated with entity perceptions (e.g., the trustworthiness of the manager) whereas entity perceptions may include cues from especially salient events. This reinforces the importance of adopting a broad and temporal approach that moves beyond an exclusive focus on event or entity-based justice rules.

### The Influence of Perspectives and Social Context

While our discussion on perspectives focused on the impact of roles, it is also important to more broadly explore how parties’ perspectives and social context can influence the motivated processes underlying fairness perceptions. Perceivers may operate from a set of implicit beliefs that can impact the cues that they perceive and make it difficult to pick up noncongruent cues. For example, some recipients may believe that people in power are generally unfair (e.g., Haselhuhn et al., 2017). This may make it difficult to perceive some cues as well as impact the dynamic between themselves and the actor, especially if the actor is in a higher power position. By contrast, some actors may be highly confident that they can manage fairness situations (e.g., they have a high core self-evaluation; Hillebrandt et al., 2021) or may be less empathetic toward the recipients (e.g., Whiteside & Barclay, 2015). These factors may make it more difficult for them to recognize some cues (e.g., emotional cues from the recipient) and/or may focus them on different motives (e.g., self-enhancement vs. relational motives).

Managing motivated processes can also involve interpersonal influence and power dynamics. For example, Cialdini and Sagarin (2005) outline six psychological processes that can motivate people to consider and be influenced by others’ perspectives, including reciprocity, social validation, commitment and consistent, friendship and liking, scarcity, and authority. This suggests that employees may be more motivated to attend to cues provided by their manager than their coworkers. Similarly, people may be less motivated to attend to cues from others when they do not expect an ongoing relationship (e.g., a manager may be less likely to attend to cues from an employee that is retiring). Asking the question of what motivates people to adopt others’ points of view or comply with their influence attempts may therefore provide an opportunity for integration with other literatures and create exciting new future research directions.

Finally, the social context may also shape motives and cues. For example, moral motives may be less influential in organizations that prioritize the bottom-line (e.g., Sherf et al., 2019). Similarly, the bias that minority leaders can encounter when enacting interpersonal justice (see Zapata et al., 2016) suggests that cues related to gender, age, and race may be differentially available or weighted depending on the organization's climate for equity, diversity, and inclusion. This suggests that it is important to further explore the factors related to perspectives and social context that can motivate and influence how people perceive fairness information.

### The Dialectic Nature of Fairness Perceptions

The MPA also highlights that fairness perceptions are socially constructed and negotiated through interactions with motivated others. The presence of others can shape which motives and cues are available. This creates exciting new research opportunities. For example, while scholars have recognized that predicaments of unfairness can emerge in which parties differ in their fairness perceptions, these predicaments have typically been attributed to an identity-based “tug of war” that can emerge as managers and employees disagree about the fairness of the manager (e.g., Whiteside & Barclay, 2015). However, the MPA highlights that these predicaments may not be caused exclusively by the manager's identity concerns or even rest primarily on the manager. Instead, predicaments of unfairness reflect differences in cue sets and/or motives from either party. These differences may also persist as employees may overweight cues that suggest the manager is unfair and underweight cues of the manager's fairness. Similarly, a manager who was previously unfair may overweight cues related to their subsequent fair behaviors to maintain their identity as a fair manager (i.e., one's own behaviors may also influence one's fairness perceptions; Hillebrandt & Barclay, 2020).

The MPA also demonstrates how the social context can shape dialectic processes (e.g., expanding or reweighting cues and/or activating motives). For example, the presence of third parties may encourage the recipient and/or actor to activate accuracy motives, which may prompt them to engage in a more thorough cue search and/or weigh cues so that their perceptions can be justified to others. In turn, this may help reduce predicaments of unfairness and/or provide the opportunity to “negotiate” fairness perceptions in a more effective manner. Similarly, the MPA also raises the interesting possibility that people may use discourse with others to manage their own fairness perceptions to reach a desired conclusion. For example, an employee who is denied a deserved promotion may feel unfairly treated by their manager but also know that they must continue working effectively with the manager. This may motivate them to change their perceptions (e.g., blame the unfairness on the situation instead of the manager) so that they can quell negative feelings about the manager and promote a positive relationship. To do so, the employee may connect with others who can provide information that can help them reach their desired conclusion (i.e., perceive the manager as fair) or engage in system justification to rationalize the unfairness (see Jost, 2019). Alternatively, an employee who wants to perceive that their manager is unfair may avoid engaging in dialectics with others who may persuade them to consider cues that will change this perception. For example, employees can find it self-threatening to receive unfavorable outcomes through fair procedures (e.g., Schroth & Shah, 2000) and may try to protect themselves by maintaining a perception that the procedure was unfair (e.g., Siegel et al., 2016), including avoiding information that challenges this perception. Thus, people can expand the scope of the event, underweight cues or the event, or avoid dialectics to manage their fairness perceptions.

Finally, while dialectic processes may seem complex to understand and manage, it is important to recognize that these processes emerge from the same underlying perceptual process. The complexity emerges from the involvement of multiple parties and the interplay between the parties. By identifying the core perceptual process (i.e., managing cue sets and motives), the MPA can make a seemingly complex situation simpler to understand and manage.

### Beyond Perceptions: A Unified and Integrated Motivated Approach to Fairness

Beyond providing an integrated approach to fairness perceptions, the MPA can also provide the foundation for unifying other fairness streams. For example, Brockner et al. (2015) differentiates nine different ways to study fairness reactions, by crossing the perspectives (i.e., recipient, actor, and third party) with the nature of fairness (i.e., perceptions of fairness, the enactment of fairness, and the desire for fairness). While Brockner et al. (2015) differentiate between these streams, the MPA provides an integration opportunity. Although we focused on the motivated nature of fairness *perceptions,* motives have also been identified for why people care about fairness (e.g., Barclay et al., 2017) and why actors adhere to justice rules (e.g., Scott et al., 2014). Consolidating these streams under a unified umbrella provided by the MPA may provide a strong and integrated theoretical foundation that can yield significant new insights. For example, the motives identified for managers’ adherence to justice rules were originally based on the aggression literature (see Scott et al., 2009). However, the MPA indicates that an expanded set of motives may be relevant for justice enactment. Further, it is important to broaden the scope of enactment to examine *fairness* rather than simply relying on the adherence to justice rules (also see Varty et al., 2021). Moreover, there can be interplay between enactment, perceptions, and desires, such as when behaviors may motivate perceptions and vice versa.

### Practical Implications

By highlighting the dynamic and dialectic nature of fairness, the MPA provides practical insights into the difficulties associated with managing fairness perceptions and how to overcome these challenges. For example, managers should recognize that their own motives may impact fairness interactions; managers may be inadvertently providing cues that undermine perceptions of fairness, narrowing the cue set that is available to the recipient, and/or creating gaps in their own self-awareness through the activation of motives that focus attention on some cues but not others. Self-reflection may be especially important to gain insight into how one's motives influence one's own and others’ behaviors and perceptions (see Barclay et al., 2017).

Beyond considering barriers and facilitators related to their own perspective, managers should also consider others’ perspectives. For example, although managers are typically advised to adhere to justice rules to enhance fairness (e.g., Skarlicki & Latham, 1996), managers can uphold justice rules and still be perceived as unfair. Thus, managers must recognize that fairness perceptions can draw on information beyond justice rules in a motivated way. Moreover, the same person may not react the same way over time to the same treatment, nor will different people necessarily react in the same way to the same treatment. This suggests that adhering to the justice rules is necessary but not sufficient. While this possibility may seem overwhelming, the MPA provides concrete strategies to actively manage fairness perceptions. By understanding that fairness perceptions are in part a function of the available cue set, managers can play an active role by ensuring that the relevant cues are available to recipients, activating accuracy motives to encourage the consideration of relevant cues, and expanding the temporal scope to include the preparation and transition stages (see Bies, 2013). For example, active dialogue (e.g., raising questions) may prompt the exchange of information and cues that can counter the other person's position and/or illuminate the validity of one's own position. Conversely, structured dialogue (e.g., being guided through a counterfactual thinking process to explore what might have been; Kray & Galinsky, 2003) or being coached to take a higher level of construal (e.g., Holt et al., 2021) can help expand the available cue set.

Importantly, these strategies may be most effective *before* the situation evolves into a predicament of unfairness in which the parties fail to negotiate. When predicaments of unfairness do arise, the MPA suggests that managers are well-served to consider how differing motives can impact fairness perceptions as well as how motives can impact how the situation unfolds and must be managed. For example, activating accuracy motives may address the differential selection and/or weighting of cues created by conflicting motives whereas providing cues that allow the recipient to have closure may allow the parties to move on from the event. Thus, recognizing the subjective and motivated nature of each of the parties involved can provide critical insights on how to effectively manage and promote fairness in the workplace.

Finally, the MPA implies that communication is key to effectively managing fairness—managers should recognize when there are discrepancies and ambiguity related to the cues and work to resolve these differences, not only during the event itself but during the broader fairness interaction. Open and trusting communication with rich communication channels (e.g., face-to-face versus email) is likely to be especially important to avoid miscommunication and enhance the cues that are available in the situation (e.g., Bies et al., 2016). Similarly, motivating people to create a shared subjective reality as well as encouraging information exchange and elaboration may be especially helpful (see Lyubykh et al., 2022). This also points to the importance of building trusting relationships and treating employees fairly on an ongoing basis (before fairness-related events occur) to facilitate these processes and guide how employees interpret subsequent fairness-related events (e.g., Barclay & Kiefer, 2019; Choi, 2008; Skarlicki et al., 2008).

## Conclusion

By showcasing the dynamic and dialectic processes underlying fairness perceptions, the MPA provides significant insights into how people perceive and experience fairness, including the challenges that can arise and how these can be overcome. Moreover, the MPA creates an exciting new research agenda for the fairness literature that encourages scholars to delve deeper into dynamic within-person processes (e.g., how people's fairness perceptions change over time) as well as the dialectic processes that can shape how fairness perceptions are socially constructed and negotiated via interactions with motivated others. The MPA also provides a strong theoretical foundation that can unify seemingly disparate research streams thereby providing the basis for a comprehensive and integrated approach to fairness. Taken together, adopting the MPA can help reinvigorate the field by providing a richer understanding and enhanced practical ability to promote fairness in the workplace.

## Notes

1. Consistent with recent distinctions, we use the term “justice” to refer to the adherence to normative rules or standards whereas “fairness” refers to subjective and evaluative judgments (see Goldman & Cropanzano, 2015).

2. To reduce complexity, our theorizing focuses on fairness perceptions without distinguishing between event-based (i.e., evaluations related to a specific situation) or entity-based perceptions (i.e., evaluations of a person that transcend specific situations; see Cropanzano et al., 2001). In the discussion, we outline how the MPA can also provide a common framework for integrating event and entity fairness perceptions.

3. While these three categories have been emphasized with the literature on fairness perceptions, other motives have been identified including a just world motive (i.e., people are motivated to perceive that people get what they deserve; Lerner, 1980) and motives related to the enactment of fairness (see Matta et al., 2020; Scott et al., 2009).

4. Whereas employees who are able to leave the organization may be motivated to select cues that indicate the organization is unfair (e.g., to justify their choice), those who must stay in the organization may be motivated to look for cues that can enable them to perceive the organization as “fair” since people generally prefer to work with systems that are perceived as “fair” to enhance their own wellbeing (e.g., system justification motives; see Proudfoot & Kay, 2014).

5. The recipient (e.g., employee) is the receiver of a decision, allocation, or treatment that can be assessed in terms of fairness. The actor (e.g., manager) is the person who enacts fairness toward the recipient. Third parties or third-party observers (e.g., colleagues) are those who may witness or have knowledge of the fairness situation but are not directly involved (e.g., Skarlicki & Kulik, 2004).
